# Engineered cancer cell membranes: An emerging agent for efficient cancer theranostics

**DOI:** 10.1002/EXP.20210171

**Published:** 2022-01-25

**Authors:** Yunqi Guo, Zhiqiang Wang, Xiangyang Shi, Mingwu Shen

**Affiliations:** ^1^ Shanghai Engineering Research Center of Nano‐Biomaterials and Regenerative Medicine, College of Chemistry, Chemical Engineering and Biotechnology Donghua University Shanghai P. R. China

**Keywords:** circulating tumor cells, functionalized cancer cell membranes, hybrid cancer cell membranes, immunity, targeting delivery

## Abstract

For efficient cancer theranostics, surface modification of nanomaterials plays an important role in improving targeting ability and reducing the non‐specific interactions with normal tissues. Recently, the biomimetic technology represented by coating of cancer cell membranes (CCMs) has been regarded as a promising method to strengthen the biocompatibility and targeting specificity of nanomaterials. Furthermore, the engineered CCMs (ECCMs) integrated with the natural biological properties of CCMs and specific functions from other cells or proteins have offered more possibilities in the field of cancer theranostics. Herein, the recent progresses in the design and preparation of ECCMs are summarized, and the applications of ECCMs in targeting delivery, activation of immunity, and detection of circulating tumor cells are reviewed. Finally, the current challenges and future perspectives with regard to the development of ECCMs are briefly discussed.

## Graphical abstract

Engineered cancer cell membranes (ECCMs) including hybrid CCMs and functionalized CCMs have offered increasing possibilities in the field of cancer theranostics. This review summarizes the recent advances in the development of ECCMs for different biomedical applications, in particular for targeted drug delivery, immunity induction, and circulating tumor cell capture and detection.

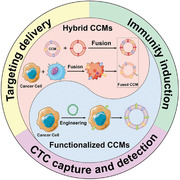

## 1 INTRODUCTION

Recently, various nanoplatforms have been developed for biomedical applications due to their good biocompatibility, high drug loading capacity, controlled drug release ability, as well as enhanced tumor penetration ability.^[^ ^1^ ^]^ These nanoplatforms including but not limited to dendrimers,^[^ ^2^ ^]^ nanogels,^[^ ^3^ ^]^ ultrasmall iron oxide nanoparticles (NPs),^[^ ^4^ ^]^ carbon dots,^[^ ^5^ ^]^ and micelles^[^ ^6^ ^]^ have been developed for cancer therapy, cancer diagnosis, or cancer theranostics. However, these nanoplatforms face a series of common problems after intravenous administration such as poor tumor targeting ability, short circulation time, and non‐specific interactions with normal tissues and so on,^[^ ^7^ ^]^ thus hindering their clinical translation. One practical method to address these issues confronted by nanomaterials is to introduce functional or active targeting molecules onto their surfaces.^[^ ^8^ ^]^ Although various ligands,^[^ ^9^ ^]^ aptamers,^[^ ^10^ ^]^ or antibodies^[^ ^11^ ^]^ have been modified onto the surfaces of nanomaterials, their applications are still impeded by the insufficient targeting efficiency and the quick clearance by reticuloendothelial system (RES).^[^ ^12^ ^]^

Fortunately, biomimetic technology has become a promising strategy adopted to decorate nanomaterials to render them with excellent biocompatibility and low immunogenicity.^[^ ^13^ ^]^ With the up‐to‐date advances in cell and molecular biology, diverse cell membranes (CMs) possessing the unique functionalities as the mostly used biomimetic materials, have exhibited significant advantages in precision cancer theranostics.^[^ ^14^, ^15^ ^]^ Since the discovery in 2011 showing that the coating of red blood cell (RBC) membranes could effectively extend the half‐life of NPs in vivo,^[^ ^16^ ^]^ natural CMs with flexible membrane proteins have been extensively used to cover NPs, thus conferring them with diverse biological interfaces.^[^ ^17^, ^18^ ^]^ Several recent reviews have summarized the applications of CM‐coated NPs in cancer theranostics, highlighting the advantages of different CMs.^[^ ^15^, ^17^, ^19^ ^]^ For example, leukocyte membranes can endow the NPs with immune escape ability,^[^ ^20^ ^]^ dendritic cell (DC) membranes render the NPs with homotypic targeting toward lymph nodes,^[^ ^21^ ^]^ and platelet membranes highly expressed with P‐selectin enable the NPs to actively target to cancer cells.^[^ ^22^ ^]^

Among the various types of CMs, cancer CMs (CCMs) with multiple tumor antigens have been widely explored for cancer theranostics.^[^ ^23^ ^]^ On the one hand, the rapid proliferative potential of cancer cells makes it much easier to obtain the CCMs than to acquire other types of CMs. On the other hand, the unique membrane proteins of CCMs enable the nanoplatforms with excellent homologous targeting to cancer cells and immune evasion from RES.^[^ ^24^ ^]^ Therefore, CCMs have been widely used to camouflage diverse NPs for different cancer nanomedicine applications such as imaging,^[^ ^25^ ^]^ drug delivery,^[^ ^26^ ^]^ and tumor vaccines.^[^ ^27^ ^]^ However, the applications of pure CCMs or pure CCMs‐derived NPs are limited because the CCMs lack the therapeutic capacity to directly treat tumors and the tumor antigens expressed on CCMs may be down‐regulated to escape from recognition of immune systems.^[^ ^28^ ^]^

**FIGURE 1:**
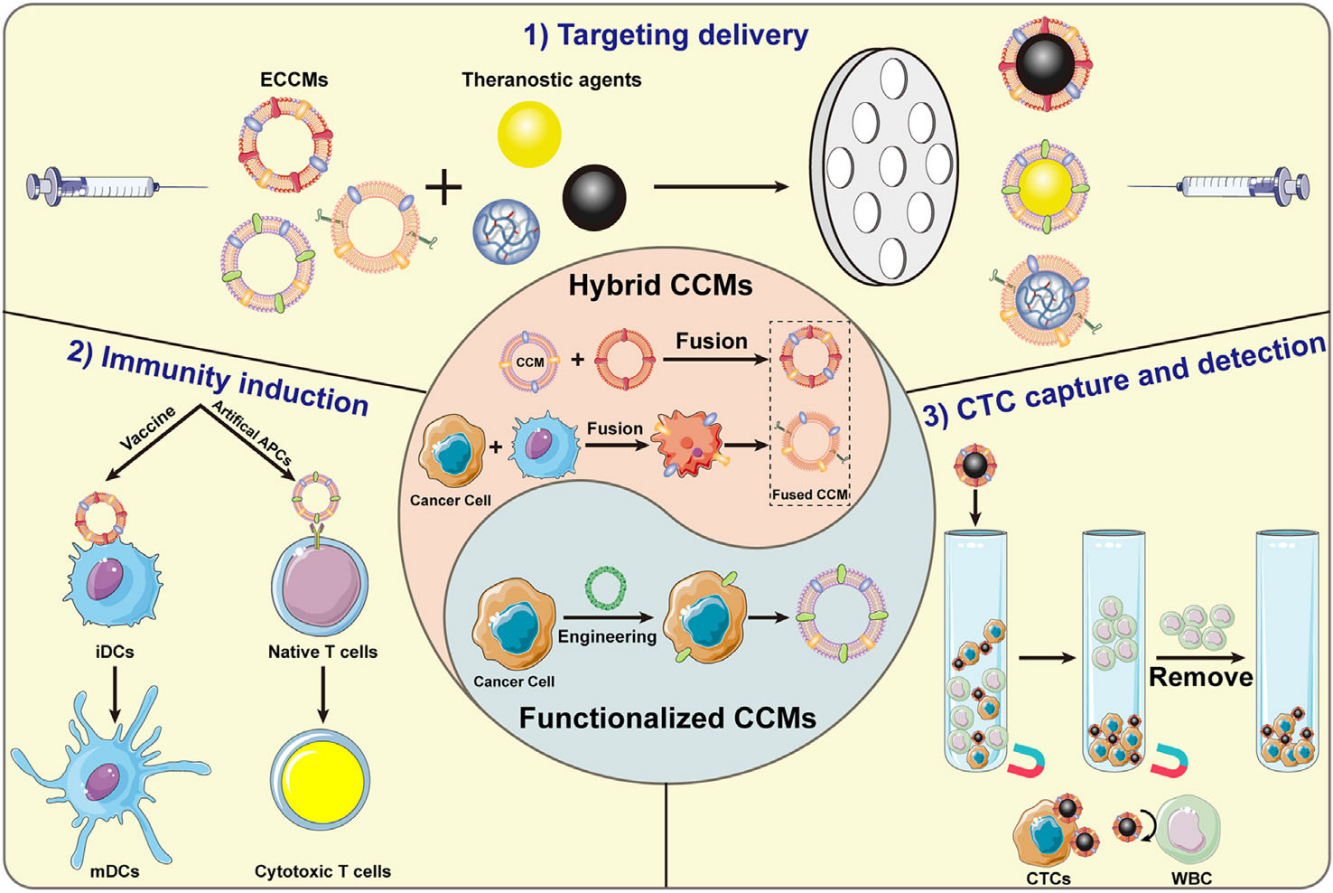
Schematic illustration of the preparation of ECCMs and their applications in cancer theranostics

Along this line, engineered CCMs (ECCMs) fabricated through fusing CCMs with other types of CMs or making CCMs express specific proteins provide a new direction to overcome the drawbacks and improve the biomedical performances of CCMs. Compared with pure CCMs, the ECCMs and ECCM‐based biomimetic nanosystems maintain the properties of CCMs and are able to undertake more complexed tasks in the tumor microenvironment.^[^ ^29^ ^]^ In particular, the beauty of ECCMs lies in the fact that they can not only be used to coat NPs for targeting delivery, but also be directly used for inducing anti‐tumor immunity utilizing the engineered membrane proteins. In this review, we introduce the recent progresses in the design and preparation of ECCMs and provide an overview of their applications in cancer theranostics, including targeting delivery, activation of immunity, and detection of circulating tumor cells (CTCs) (Figure 1). The representative preparation methods, applications, and advantages of various ECCMs are summarized in Table 1. We also discuss the current challenges and future perspectives of ECCMs in order to further enhance their functions and efficacy. It should be noted that this is not a comprehensive review of full aspects of the ECCMs, but rather to discuss some key progresses in ECCMs for cancer theranostics over the past five years.

**TABLE 1:** Representative preparation methods, applications, and advantages of various ECCMs

**Preparation method**	**Source**	**Applications**	**Advantages**	**Ref**.
Fusion before extracting cell membrane	4T1 cells + Dendritic cells (DCs)	Tumor vaccine	Induce anti‐tumor immune response	^[^ ^30^ ^]^
		Artificial antigen‐presenting cells (APCs)	Directly stimulate native T cells; home to the lymph node	^[^ ^31^ ^]^
Fusion after extracting cell membrane	4T1 cells + RAW 264.7 cells	Targeting delivery	Multi‐targeting capability; accumulation at inflammation sites	^[^ ^32^ ^]^
	MCF‐7 cells + RAW 264.7 cells	Capture and detection of CTCs	Increase the affinity; reduce the interference	^[^ ^33^ ^]^
	B16‐F10 cells + RBCs	Targeting delivery	Homotypic targeting; long blood circulation	^[^ ^34^ ^]^
		Tumor vaccine	Deliver tumor‐associated antigens; target APCs in spleen	^[^ ^35^ ^]^
	B16‐F10 cells + Gram‐negative bacterial	Targeting delivery and tumor vaccine	Homologous targeting; immune response induction	^[^ ^36^ ^]^
Functionalized CCMs	4T1 cells + KillerRed plasmid	Targeting delivery	Expression of the photosensitizer KillerRed protein on CMs; homologous targeting	^[^ ^37^ ^]^
	B16 cells + Mannose moiety	Tumor vaccine	Deliver tumor‐specific antigens; enhance APC uptake	^[^ ^38^ ^]^
	B16 cells + plasmid encoding CD80	Artificial APCs	Directly stimulate native T cells	^[^ ^39^ ^]^

## 2 PREPARATION of ENGINEERED CANCER CELL MEMBRANES

The engineering of CCMs makes it possible to integrate the applicable membrane proteins of different CMs or insert the additional functional proteins into the CCMs. Based on the strategy to prepare ECCMs, the ECCMs can be divided into two types: Hybrid CCMs and functionalized CCMs.

### 2.1 Hybrid cancer cell membranes

The most convenient method to prepare ECCMs is to blend CCMs with membrane fragments from other types of cells to form hybrid CCMs, which can optimize the construction of membrane proteins and inherit the intrinsic properties of source cells.^[^ ^40^ ^]^ Since the CCMs are characterized by homologous targeting and immune escape ability, they can be fused with different types of CMs to strengthen their performance in cancer theranostics, for example, with erythrocyte membranes to decrease the clearance by RES^[^ ^41^ ^]^ or leukocyte membranes to reduce the interference of homologous leukocytes.^[^ ^33^ ^]^ Depending on the time of membrane fusion, the preparation of hybrid CCMs can be categorized into two approaches. The first approach is to fuse cancer cells with another type of cells to form fused cell lines before extracting the hybrid CCMs, and the second one is to extract CCMs and another type of CMs and fuse the two types of membranes to obtain the hybrid CCMs.

#### 2.1.1 Fusion before extracting cell membranes

**FIGURE 2:**
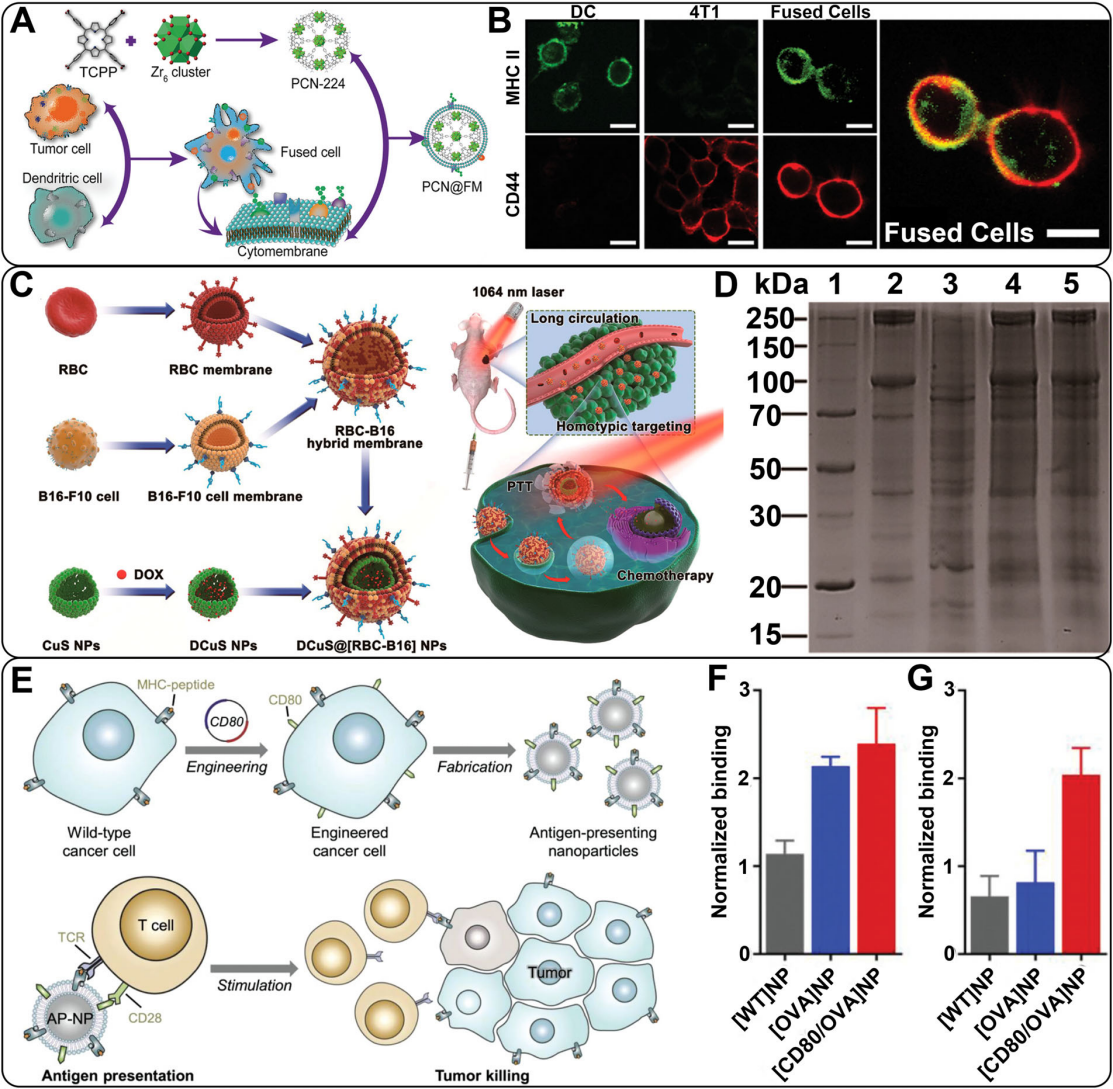
(A) Preparation of fused CM‐coated NPs. (B) Confocal microscopic observation of DCs, 4T1 cells, and fused cells co‐stained with MHC II‐fluorescein isothiocyanate antibody (green) and CD44‐allophycocyanin antibody (red). Scale bar = 16 μm. Reproduced with permission.^[^ ^30^ ^]^ Copyright 2019, Wiley‐VCH. (C) Preparation of DCuS@[RBC‐B16] NPs and the synergistic photothermal/chemotherapy of melanoma. (D) SDS‐PAGE protein analysis of different materials (1: marker, 2: RBC membranes, 3: B16‐F10 CMs, 4: RBC‐B16 CMs, and 5: DCus@[RBC‐B16] NPs). Reproduced with permission.^[^ ^37^ ^]^ Copyright 2018, American Chemical Society. (E) Schematic illustration of functionalized CCM‐coated NPs for direct antigen presentation. Relative binding of antibodies against (F) Kb‐SIINFEKL and (G) CD80 to [WT]NPs, [OVA]NPs, and [CD80/OVA]NPs, respectively (*n* = 3, mean ± SD). Reproduced with permission.^[^ ^39^ ^]^ Copyright 2020, Wiley‐VCH

In this way, cancer cells and another cell lines are required to be co‐cultured together to form fused cell lines that share and possess the characteristics of two source cells. The specific membrane proteins of the original cells can be presented on the membrane of the fused cells. Therefore, the hybrid CCMs extracted from the fused cells have at least two types of biological functions. For instance, Liu and coworkers fused the murine mammary carcinoma tumor (4T1) cells and DCs under the stimulation of polyethylene glycol (PEG) to obtain the fused cells and further extracted the hybrid CCMs (Figure 2A).^[^ ^30^ ^]^ Briefly, 4T1 cells were mixed with DCs at a ratio of 1:2 in the phosphate buffer saline (PBS) containing PEG and dimethyl sulfoxide to fuse the two cells. Then, the fused cells were incubated with interleukin (IL)‐4 for additional 6 h before harvesting the hybrid membranes to ensure that the co‐stimulatory molecules and peptides‐major histocompatibility complex (MHC) from DCs were expressed on the membranes of fused cells. Different from the pure cancer cells that down‐regulate related antigens to achieve immune escape, the complete tumor antigens as well as DC‐derived immune stimulatory molecules were present on the membranes of the fused cells. As shown in Figure 2B, the fused cells exhibit both CD44 (from 4T1 cells) and MHC II (from DCs) on the cell surface, demonstrating the successful formation of fused cells. Therefore, with the help of tumor antigens and MHC II, the membranes of the fused cells could enhance the recognition by immune cells and efficiently induce immune response to inhibit the growth of tumors. However, the applications of this method to prepare ECCMs are limited due to the complicated purification steps and the possibility to form unwanted fusion cell lines.

#### 2.1.2 Fusion after extracting cell membranes

The other approach is to fuse extracted CCMs and other types of CMs to obtain the hybrid CCMs with more precise structure and protein composition. This kind of hybrid CCMs show the inherent properties of at least two source CMs. The fusion of extracted CMs can be achieved through a relatively simple operation, such as, stirring and sonication.^[^ ^42^ ^]^ For instance, Wang et al. developed the RBC‐B16 hybrid membranes through the fusion of RBC membranes and B16‐F10 CMs (Figure 2C).^[^ ^34^ ^]^ In this case, the RBC membranes and B16‐F10 CMs were collected respectively through gradient centrifugation before fusing. Subsequently, the RBC membranes were mixed with the B16‐F10 CMs at a mass ratio of 1:1 under sonication for 10 min to accomplish the fusion process. As shown in Figure 2D, the sodium dodecyl sulfate‐polyacrylamide gel electrophoresis protein analysis showed that the RBC‐B16 hybrid membranes (Line 4) exhibited characteristic proteins of both RBC membranes (Line 2) and B16‐F10 CMs (Line 3) on the gel, suggesting the successful fusion of the two types of CMs. Therefore, the obtained hybrid CCMs could render the NPs with characteristic functionality of both RBC membranes and B16‐F10 CMs, including prolonged blood circulation time and improved homologous targeting ability.

### 2.2 Functionalized cancer cell membranes

Since the functions of hybrid CCMs only stem from the membrane proteins of source cells, it is necessary to introduce molecules or proteins with special properties into CCMs, thus expanding the application scope of CCMs. Transfection of specific genes into cancer cells is one commonly used method to prepare functionalized CCMs. Recently, Jiang et al. engineered wild‐type (WT) B16‐F10 cells to overexpress two additional genes (Figure 2E).^[^ ^39^ ^]^ The first gene was a cytosolic form of ovalbumin (OVA), which was selected as the model antigen to promote immunity. The second one was a plasmid DNA encoding CD80, a co‐stimulatory marker that can engage the CD28 receptor on T cells. By this way, the functionalized CCMs derived from the double knock‐in B16‐CD80/OVA cells could be used for coating NPs to construct artificial APCs. The NPs camouflaged by the functionalized CCMs ([CD80/OVA]NP) exhibited the highest binding efficiency to both SIINFEKL peptide bound to H‐2Kb (Kb‐SIINFEKL, a representative OVA peptide) antibody and CD80 antibody, demonstrating the successful fabrication of CD80/OVA‐functionalized CCMs (Figure 2F,G).

In another work, Kim et al. synthesized the functionalized CCMs having KillerRed (KR) protein, a photosensitizer with red fluorescence, embedded on their surface.^[^ ^37^ ^]^ In their approach, 4T1 cells were transfected with KR‐expressing plasmid DNA encoding a membrane‐localization signal peptide to have KR proteins immobilized on the 4T1 CMs. The successful expression of KR proteins was confirmed through detection of the KR‐related red fluorescence and the transfection efficiency was measured to be 90.5%. Then, the KR protein‐functionalized CCMs were extracted and hybridized with lipid adjuvant and helper lipids to obtain the functionalized CCM‐based lipocomplex for targeted tumor photodynamic therapy.

## 3 ENGINEERED CANCER CELL MEMBRANES FOR CANCER THERANOSTICS

Considering the original characteristics of CCMs, the most important application of ECCMs is to realize the effective diagnosis and treatment of tumors. ECCMs have been widely developed to camouflage various NPs with theranostic properties through physical extrusion,^[^ ^43^ ^]^ sonication,^[^ ^44^ ^]^ or microfluidics combined with electroporation.^[^ ^45^ ^]^ The physical extrusion method utilizes porous polycarbonate membranes to let the ECCMs cover onto the surface of NPs through mechanical forces, which can make full use of ECCMs to produce ECCM‐coated NPs. However, the low output efficiency of physical extrusion affects their further applications. The sonication method employs ultrasonication energy to facilitate the coating of ECCMs and the coating efficiency can be well‐controlled by the sonication time, power, or frequency. However, the sonication may lead to uneven distribution of ECCMs on the surface of NPs. As for the method of microfluidics combined with electroporation, the ECCMs and NPs are completely fused in a microfluidic system to produce ECCM‐coated NPs, and the critical issue is how to effectively maintain the activity of membrane proteins during the microfluidic synthesis. ECCM‐coated NPs synthesized using different methods can be used for different biomedical applications, in particular targeted drug delivery, tumor vaccine, and detection of CTCs. In addition to the synthesis of ECCM‐coated NPs, ECCMs themselves can also be directly used as therapeutic agents to treat tumors through the use of their own unique properties.

### 3.1 Targeting delivery

Various drug‐loaded NPs^[^ ^46^ ^]^ have been developed to tackle cancer, however their functionalities cannot be fully realized due to their poor cancer targeting ability. The combination of CCMs with drug delivery nanosystems benefits the specific targeting delivery due to the prominent features of CCMs of homologous targeting and immune escape.^[^ ^47^ ^]^ Furthermore, the ECCMs can expand the functions and scope of the membrane‐camouflaged delivery nanosystems.

**FIGURE 3:**
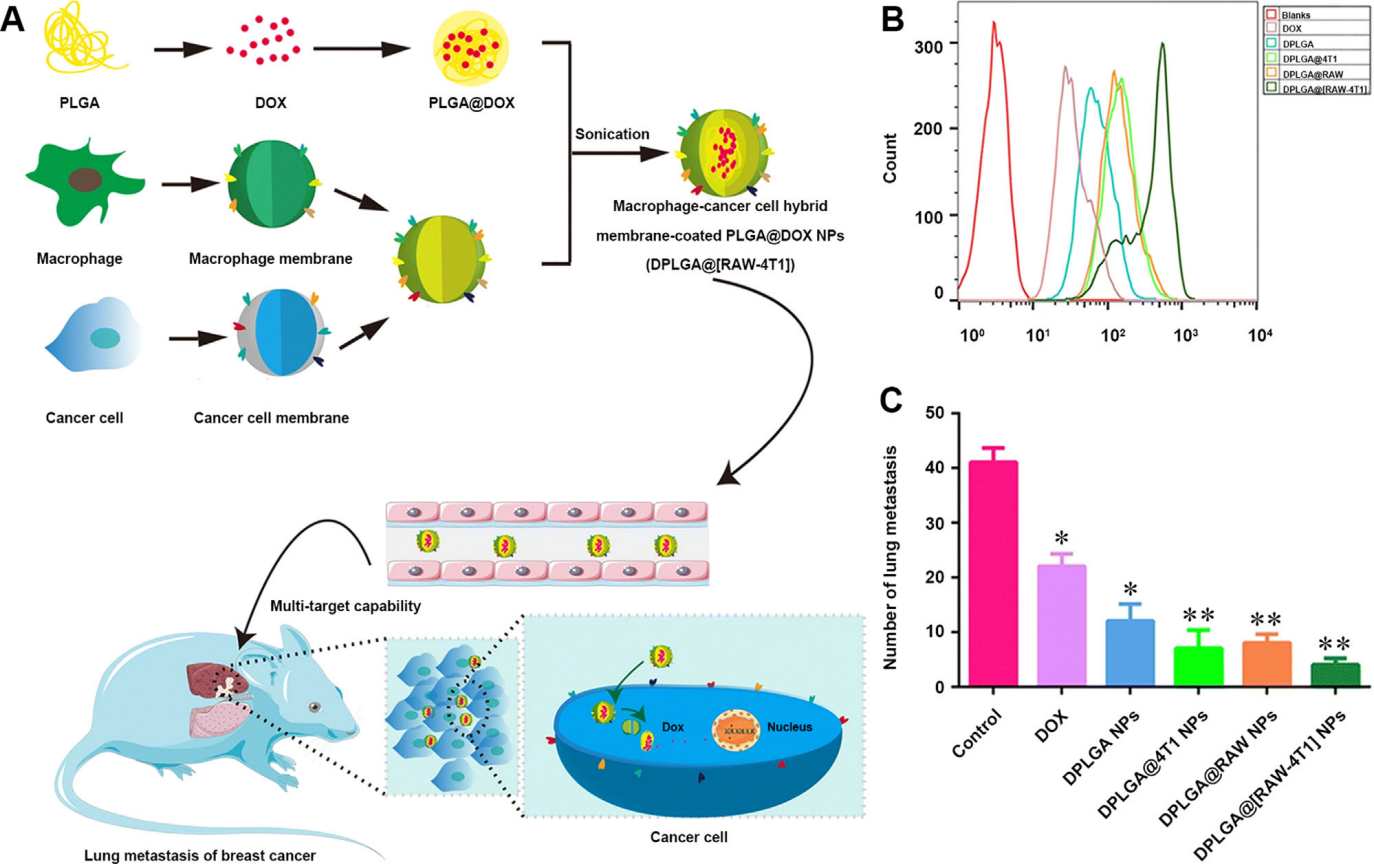
(A) Formation and application of DPLGA@[RAW‐4T1] NPs. (B) Flow cytometry analysis of 4T1 cell apoptosis after incubation with free DOX, DPLGA NPs, DPLGA@4T1 NPs, DPLGA@RAW NPs, and DPLGA@[RAW‐4T1] NPs, respectively for 48 h. (C) Quantitative analysis of lung metastatic nodules in each group. Reproduced with permission.^[^ ^32^ ^]^ Copyright 2020, Springer Nature

In a recent work, Gong et al. synthesized doxorubicin (DOX)‐loaded poly(lactic‐*co*‐glycolic acid) (PLGA) NPs and prepared hybrid CCMs through fusing cell membrane components from macrophages (RAW 264.7 cells) and 4T1 cells, then covered the DOX‐loaded PLGA NPs with the hybrid CCMs (Figure 3A).^[^ ^32^ ^]^ The macrophage membranes were introduced since they are present in the early diffusion of cancer metastasis, and can bind to the metastatic cancer cells through the expressed integrin α4 and β1.^[^ ^48^ ^]^ Through integration of the two types of CMs, the prepared hybrid ECCM‐coated DOX‐loaded PLGA NPs (DPGA@[RAW‐4T1] NPs) exhibited specific targeting not only to the homologous cancer cells, but also to the metastasis cancer cells, realizing treatment of both cell types. The DPGA@[RAW‐4T1] NPs were first used to treat 4T1 cells in vitro to induce the highest percentage of cell apoptosis (58.15%) than free DOX and single CM‐coated NPs (Figure 3B), then further used to treat a breast tumor model with lung metastasis in vivo to exhibit a much better anti‐metastatic activity than the single CM‐coated NPs (Figure 3C). In general, the fusion of macrophage membranes and CCMs provides a promising strategy to realize the targeting delivery of drug‐loaded nanoplatforms to both the primary tumor and the metastasis tumor for efficient therapy.

In another work, Xie et al. developed a drug delivery system based on hybrid CCM‐functionalized liposomes that were loaded with the cytotoxic compound chikusetsusaponin IVa methyl ester (CSME) and photosensitizer chlorin e6 (Ce6) for combinational therapy.^[^ ^49^ ^]^ In their approach, the hybrid CCMs derived from RBCs and 4T1 cells were fused into the CSME‐loaded liposomes under sonication to confer the liposomes with specific targeting ability and extended blood circulation time. Subsequently, the Ce6 was loaded into the bilayer and cavity structure of the liposomes, followed by further modification of Arg‐Gly‐Asp (RGD) onto the liposome surface to strengthen their targeting ability. The hybrid CCM‐functionalized liposomes can significantly improve the water solubility, biocompatibility, and targeting ability of both CSME and Ce6, and display less uptake by macrophages and more uptake by 4T1 cells than those without membrane fusion. The half‐decay time of the hybrid CCM‐functionalized liposomes was 3.5 h, which was significantly longer than that of free Ce6 (1.3 h) and liposomes without membrane fusion (1.9 h). Both the in vivo fluorescence imaging and anti‐tumor assay data demonstrated that the fabricated liposomes could be significantly accumulated at the tumor site to realize improved synergistic therapeutic effect.

### 3.2 Induction of immunity

Since the immune system plays an essential role in protecting body health and eliminating harmful cells, immunotherapy that can simulate the immune system has become one promising method for tumor therapy. To induce anti‐tumor immunity, it is important to utilize tumor antigens and stimulatory molecule to activate the immune cells and reverse the immunosuppression.^[^ ^50^ ^]^ During the process of immunotherapy, the tumor‐associated antigens can be recognized and processed by APCs that can present the specific antigens to native T cells to activate cytotoxic T lymphocytes (CTLs) for cancer cell treatment.^[^ ^51^ ^]^ Therefore, the CCMs with multiple tumor antigens have a great potential in improving the response of immune system, and the ECCMs with more special proteins can be designed as tumor vaccines to promote the maturation of APCs or artificial immune cells to directly activate native T cells.

#### 3.2.1 Tumor vaccine

Tumor vaccines that use tumor antigens and immune adjuvants to stimulate the APCs and induce anti‐tumor immunity have aroused extensive attention in recent years.^[^ ^52^ ^]^ Owing to the fact that the CCMs only maintain part of tumor antigens, the effect of CCM‐derived tumor vaccines is limited without the participation of immune adjuvants. Therefore, tumor vaccines can be developed using CCM‐coated NPs, where the NPs act as adjuvants to facilitate the activation of immune cells.^[^ ^53^ ^]^ It seems to be a promising way to prepare tumor vaccines using natural immune adjuvants to engineer the CCMs.^[^ ^54^ ^]^

**FIGURE 4:**
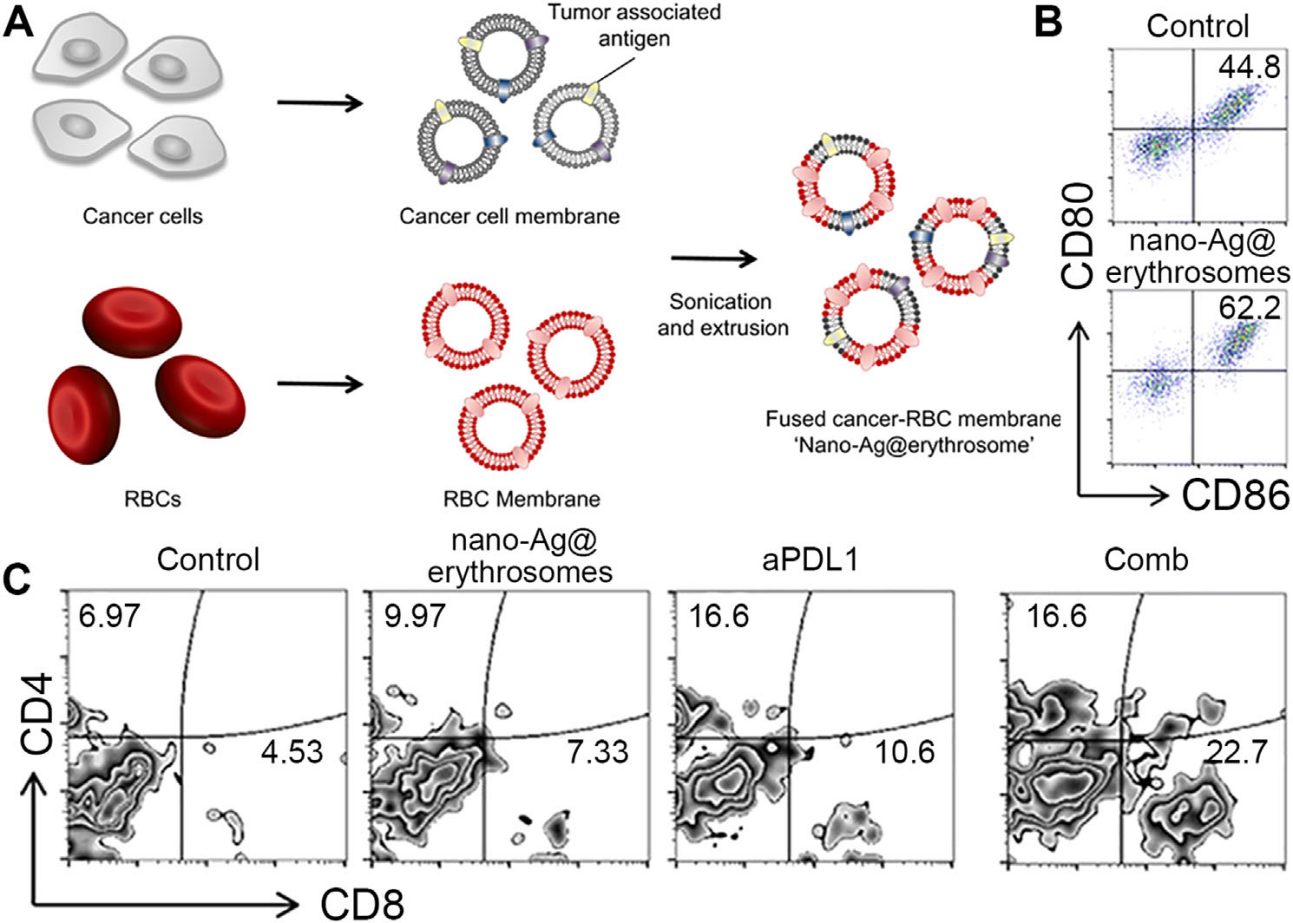
(A) Preparation of nano‐Ag@erythrosomes. (B) Flow cytometric analysis of maturation of DCs after treated with nano‐Ag@erythrosomes for 12 h. (C) Flow cytometric analysis of the number of CD4^+^ and CD8^+^ T cells in a percentage of the total CD45^+^ cell population in the tumor after different treatments. Reproduced under the terms of the Creative Commons Attribution‐NonCommercial license.^[^ ^35^ ^]^ Copyright 2019, American Association for the Advancement of Science

Encouraged by the ability of senescent RBCs to target splenic APCs, Han et al. fused B16‐F10 CMs with RBC membranes to develop tumor antigen‐loaded nanoerythrosomes (nano‐Ag@erythrosome) as a tumor vaccine (Figure 4A).^[^ ^35^ ^]^ Herein, the RBC membranes can be considered as a natural immune adjuvant, which utilizes their splenic APC targeting capacity to deliver the tumor antigens on the B16‐F10 CMs to APCs. The ratio of RBC membranes to B16‐F10 CMs was optimized to be 20: 1 to render the nano‐Ag@erythrosomes with the best targeting specificity to the spleen. The treatment of nano‐Ag@erythrosomes induced 62.2% maturity of DCs in vitro (Figure 4B) and significantly activated CTLs in vivo. Hence, the developed nano‐Ag@erythrosomes could effectively deliver tumor‐associated antigens to APCs in spleen, promote the maturation of APCs, and further activate native T cell to inhibit tumor growth. To overcome the immunosuppression of tumors and enhance the proliferation of T cells, the nano‐Ag@erythrosomes were further combined with anti‐programmed death 1 ligand 1 (aPDL1) for immune checkpoint blockade therapy. As shown in Figure 4C, the mice treated with the vaccine and aPDL1 resulted in the higher percentage of tumor infiltrating CD8^+^ T cell (22.7%) than those only treated with the vaccine (7.33%), demonstrating that the combination of the vaccine and aPDL1 could efficiently activate the immune system for enhanced tumor inhibition.

Bacterial outer membrane vehicles (OMVs) with many antigens can also be considered as a prospective natural adjuvant to induce immune response.^[^ ^55^ ^]^ Furthermore, the OMVs can promote the secretion of antitumor cytokines, including interferon γ (IFN‐γ), tumor necrosis factor α, and IL‐12.^[^ ^56^ ^]^ In a recent work, Wang et al. prepared the hybrid CCMs consisting of B16‐F10 CMs and OMVs derived from *E. coli DH5α* to strengthen the anti‐tumor immune response.^[^ ^36^ ^]^ The hybrid CCMs were used to camouflage hollow polydopamine (HPDA@[OMV‐CC]) NPs for photothermal therapy of tumors. The addition of OMVs significantly promoted the maturation of DCs as the percentage of CD80^+^ or CD86^+^ DCs after treated with the HPDA@[OMV‐CC] NPs significantly increased to 66.1% and 84.2%, respectively. The enhanced levels of IL‐12 and IFN‐γ in the tumor tissues further demonstrated that the HPDA@[OMV‐CC] could promote the secretion of cytokines and activate immune response. In summary, the hybrid CCM‐coated nanoplatform could act as a tumor vaccine to induce specific anti‐tumor immunity and realize the homologous targeting delivery of therapeutic agents in vivo.

#### 3.2.2 Artificial APCs

Different from induction of anti‐tumor immunity via promoting the maturation of DCs, the other important approach to activate immune system is to directly stimulate native T cells, thus producing CTLs. The APCs always play the role of presenting the tumor antigens to native T cells through expressed p‐MHC or co‐stimulatory molecules on the cell surface. Inspired by the antigen presentation mechanism of APCs, various artificial APCs that can replace the functions of endogenous APCs have been designed for presenting antigens to native T cells. Cancer cells that overexpress MHC on the cell membranes have been used to prepare CCM‐based artificial APCs to directly stimulate the population of cancer‐targeting CTLs.^[^ ^57^ ^]^

As mentioned in Section 2.2, the WT‐B16 cells were engineered to express OVA and CD80.^[^ ^39^ ^]^ Therefore, the membranes of the double knock‐in B16 CD80/OVA cells expressing CD80, OVA, as well as MHC have been used to construct artificial APCs. In this context, the functionalized CCMs were collected and coated onto PLGA NPs under sonication to construct the biomimetic artificial APCs ([CD80/OVA]NPs). The obtained [CD80/OVA]NPs with a size of 100 nm were able to successfully stimulate T cells. In vitro biological activity assay showed that only the double knock‐in [CD80/OVA]NPs could elicit the phenotypic changes of T cells and activate multiple CD8^+^ T cell phenotypes, including CD69^+^, CD25^+^, and memory T cells. The [CD80/OVA]NPs showed significant inhibitory effect in the treatment of either prophylactic B16 or therapeutic B16 tumor model and significantly improved the survival rate of tumor‐bearing mice. In summary, the [CD80/OVA]NPs can act as artificial APCs to directly stimulate T cells, thus inducing anti‐tumor immune response to inhibit tumor growth.

**FIGURE 5:**
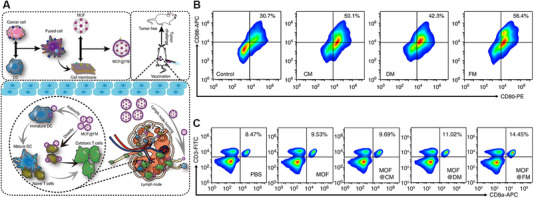
(A) Preparation of MOF@FM and the mechanism of MOF@FM to prevent tumor growth. (B) Flow cytometry analysis of the maturation of DCs after incubated with CM, DM, and FM for 48 h (*n* = 5, mean ± SD). (C) Flow cytometry analysis of CD3^+^ and CD8^+^ splenic lymphocytes at 7 days post immunization twice (*n* = 3, mean ± SD). Reproduced with permission.^[^ ^31^ ^]^ Copyright 2019, Springer Nature

In another work, Zhang's group acquired the fused membranes (FMs) from the fused cells composed of 4T1 cells and DCs, and used the FMs to camouflage a fluorescent metal‐organic framework (MOF) to achieve the MOF‐supported FM (MOF@FM).^[^ ^31^ ^]^ The FMs with whole tumor antigens and immunological co‐stimulatory molecules enabled the MOF@FM to act as a tumor vaccine to be recognized by DCs or act as artificial APCs to directly stimulate T cells (Figure 5A). As shown in Figure 5B, the treatment of FMs led to formation of 56.4% CD80^+^ and CD86^+^ DCs in vitro, which was higher than PBS group (30.7%), pure 4T1 CMs (CM) group (50.1%), and pure DCs membranes (DM) group (42.3%). The percentage of CD80^+^ and CD86^+^ DCs after treated with CM was higher than that treated with DM, demonstrating that the CM carrying tumor antigens played a leading role in FMs‐induced maturation of DCs. The splenocytes of mice treated with MOF@FM exhibited higher population of CD3^+^ and CD8^+^ T cells (14.45%) than those treated with other groups of MOF@CM (9.69%) and MOF@DM (11.02%) (Figure 5C). These results suggest that the co‐stimulatory molecules from DM allow the MOF@FM to function like APCs to simulate native T cells. With the combination of the two pathways to induce immune response, the MOF@FM exhibited the best tumor‐prevention effect in vivo.

### 3.3 Capture and detection of circulating tumor cells

The CTCs in peripheral blood circulatory system originating from solid tumors are closely related to the development, metastasis, and recurrence of tumors.^[^ ^58^ ^]^ Therefore, it is of great significance to develop new biological technology for efficient isolation and detection of CTCs in blood. Recently, NP‐based sensors with a large surface area to volume ratio have been widely researched to capture and detect CTCs.^[^ ^59^ ^]^ However, the number of CTCs in the whole blood is extremely low compared with billions of other types of cells, such as RBCs and white blood cells (WBCs).^[^ ^60^ ^]^ Therefore, the detection efficiency of CTCs is always limited by the interference from RBCs or WBCs.^[^ ^61^ ^]^ With the homologous targeting ability of CCMs, the CCM‐coated NPs were demonstrated to recognize CTCs in blood. In particular, the ECCM‐derived sensors may have improved targeting ability toward CTCs and reduced interference from other types of cells, thus enhancing the separation purity of CTCs.

**FIGURE 6:**
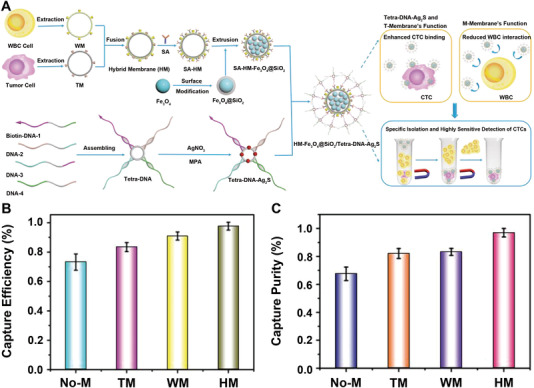
(A) Preparation of HM‐Fe_3_O_4_@SiO_2_/Tetra‐DNA‐Ag_2_S nanoplatform for the isolation and detection of CTCs. (B) Capture efficiency based on the statistics of 500 MCF‐7 cells labeled with different probes (No‐M/TM/WM/HM‐Fe_3_O_4_@SiO_2_/Tetra‐DNA‐Ag_2_S) in 10^6^ RAW264.7 cells. (C) Capture purity based on the statistics of 500 MCF‐7 cells incubated with different probes (No‐M/TM/WM/HM‐Fe_3_O_4_@SiO_2_/Tetra‐DNA‐Ag_2_S) and mixed with the background cells of 10^6^ RAW264.7 cells and 10^6^ Hela cells. Reproduced with permission.^[^ ^33^ ^]^ Copyright 2020, Wiley‐VCH

For instance, Ding et al. developed a nanoplatform for the capture and detection of CTCs by the combination of multivalent aptamer‐functionalized Ag_2_S nanodots (NDs) with hybrid CCM‐camouflaged magnetic NPs (Figure 6A).^[^ ^33^ ^]^ In brief, CCMs (from MCF‐7) and WBC membrane (from RAW 264.7 cells) were fused to obtain the hybrid CCMs (HM). The HM‐coated magnetic NPs were further modified with streptavidin (SA), then grafted with the aptamer‐functionalized Ag_2_S NDs through SA‐biotin interaction. Herein, the multivalent aptamers (Tetra‐DNA) were first prepared by DNA assembly and further mixed with the precursor solution of Ag_2_S NDs, then the mixture solution was heated to 60℃ to synthesize the multivalent aptamer‐functionalized Ag_2_S NDs via a one‐pot method. The multivalent aptamers could further increase the affinity between the prepared bioprobe and CTCs. As expected, the prepared HM‐coated magnetic NPs displayed enhanced separation performance of CTCs due to their homotypic binding to CTCs with reduced interference from WBCs. In mixed cell samples (500 MCF‐7 cells and 10^6^ RAW264.7 cells), the capture efficiency of the prepared bioprobe for CTCs was 97.63%, which was higher than that of uncoated bioprobe (No‐M), pure CCM‐coated bioprobe (CM), or pure WBC membrane‐coated bioprobe (WB) (Figure 6B). The HM‐coated bioprobe also had the best capture purity (96.96%) among all groups due to the specific CTC binding and reduced WBC interference (Figure 6C). The HM‐coated bioprobe exhibited excellent average recovery rates of the spiked cells (MCF‐7 cells) in lysed blood (96.24%) and whole blood (90.25%). In summary, the combination of HM with multivalent aptamer resulted in the sensitive and specific detection of CTCs.

## 4 CONCLUSIONS AND PERSPECTIVES

Remarkable advances have been made in the development of ECCMs and ECCM‐coated nanoplatforms to facilitate cancer theranostics. This review mainly summarized the preparation methods of ECCMs and the applications of ECCMs in cancer theranostics. Various methods have been investigated to prepare ECCMs, including fusion of cells, fusion of CMs, and functionalization of CCMs. Encouraged by the diverse functions of ECCMs, ECCMs and ECCM‐derived nanoplatforms have been developed for targeting delivery, activation of immunity, and detection of CTCs.

Despite such desired advances made in the preparation and application of ECCMs, there are still several challenges remaining to be explored. First, it will make a lot of sense to let the ECCMs retain the required membrane proteins during the synthetic process, which is meaningful to guarantee the functions of ECCMs. Second, it is important to optimize the functionalization approach of ECCMs to prevent the damage of the original membrane structure and proteins. Third, considering that the ECCMs carry various membrane proteins and tumor antigens, the in vivo long‐term biosafety of ECCMs should be investigated and ways to improve their biosafety should be explored. To sum up, the inherent characteristics of ECCMs make them exhibit great potentials in precision cancer theranostics, and the opportunities and challenges confronted by the ECCMs will further promote the continuous exploration of ECCMs in the field of biomedicine.

## CONFLICT OF INTEREST

There are no conflicts of interest to declare.
